# Electrospun Zein Fibers Incorporating Poly(glycerol sebacate) for Soft Tissue Engineering

**DOI:** 10.3390/nano8030150

**Published:** 2018-03-08

**Authors:** Lena Vogt, Liliana Liverani, Judith A. Roether, Aldo R. Boccaccini

**Affiliations:** 1 Institute of Biomaterials, Department of Materials Science and Engineering, University of Erlangen-Nuremberg, Cauerstr. 6, 91058 Erlangen, Germany; lena.vogt@fau.de; 2 Institute of Polymer Materials, Department of Materials Science and Engineering, University of Erlangen-Nuremberg, Martensstr. 7, 91058 Erlangen, Germany; judith.roether@fau.de

**Keywords:** zein, poly(glycerol sebacate), electrospinning, nano-fibers, soft tissue engineering

## Abstract

For biomedical applications such as soft tissue engineering, plant proteins are becoming increasingly attractive. Zein, a class of prolamine proteins found in corn, offers excellent properties for application in the human body, but has inferior mechanical properties and lacks aqueous stability. In this study, electrospun scaffolds from neat zein and zein blended with prepolymer and mildly cross-linked poly(glycerol sebacate) (PGS) were fabricated. Less toxic solvents like acetic acid and ethanol were used. The morphological, physiochemical and degradation properties of the as-spun fiber mats were determined. Neat zein and zein-PGS fiber mats with high zein concentration (24 wt % and 27 wt %) showed defect-free microstructures. The average fiber diameter decreased with increasing PGS amount from 0.7 ± 0.2 µm to 0.09 ± 0.03 µm. The addition of PGS to zein resulted in a seven-fold increase in ultimate tensile strength and a four-fold increase in failure strain, whereas the Young’s Modulus did not change significantly. Degradation tests in phosphate buffered saline revealed the morphological instability of zein containing fiber mats in contact with aqueous media. Therefore, the fibers were in situ cross-linked with *N*-(3-Dimethylaminopropyl)-*N*′-ethylcarbodiimide (EDC)/*N*-Hydroxysuccinimide (NHS), which led to improved morphological stability in aqueous environment. The novel fibers have suitable properties for application in soft tissue engineering.

## 1. Introduction

Until now, the regeneration and reconstruction of complicated three-dimensional soft tissues, e.g., cardiovascular or adipose tissue, is challenging and problematic. Current reconstructive treatments and procedures such as the relocation of soft tissues from one area of the body to another area, are severely limited by donor site morbidity and malformation, complications from potential toxicity of implants and foreign body response, and unsatisfactory and unpredictable results [1,2]. New strategies, like tissue engineering try to tackle these drawbacks. Among the three main factors, scaffolds, cells and growth factors, which are crucial for the success of a tissue engineered product, scaffolds play a key role from an engineering point of view [3,4]. The developed scaffolds should closely imitate the components and structural aspects of the extracellular matrix (ECM), be biocompatible as well as biodegradable and should offer adequate morphological, mechanical and degradation properties [3,4]. To mimic the native fibrous ECM structure, which is composed of various interwoven protein fibers with diameters ranging from tens to hundreds of nanometers [3,4], several fabrication techniques such as electrospinning, phase separation and self-assembly have been developed [3]. The highly versatile technique of electrospinning has attracted significant attention in the last years due to its ability to generate fibers similar to the fibrous structures of the native ECM, its processability of a wide range of materials, the straightforward nature of the process and its cost-effectiveness [3].

As materials for tissue engineering applications, proteins have gained increasing interest and are a convenient alternative to carbohydrates and synthetic polymers due to their abundance in the human body, their bio- and cyto-compatibility as well as their easy maintenance of the functions of the ECM, in contrast to synthetic polymers, which lack functional groups [5]. In the broad field of proteins, plant proteins such as zein, soy protein, gliadin, gluten or glutenin, show promising properties for applications in medicine [5]. In comparison to animal proteins like bovine collagen, plant proteins are readily available, being a co-product when cereal grains are processed for food or fuel, these proteins are biodegradable and have considerably less potential to be immunogenic [5]. Furthermore, due to the presence of amino and carboxyl groups, proteins carry different charges depending on the pH, which makes them attractive for the delivery of various types of pharmaceuticals [5]. One of the main plant proteins is the corn prolamin protein called zein [6,7]. Zein, the major storage protein in the endosperm of corn, is classified into three different fractions, based on differences in solubility and molar mass: α-, β- and γ-zein [8]. It has been shown to be resistant to microbial attacks, it has low toxicity, is biologically compatible, biodegradable, renewable and FDA approved as one of the recognized, safe excipients for film coatings of pharmaceuticals [8,9,10,11,12]. Zein, which is alcohol soluble, has an amphiphilic polymeric nature, which makes it easily joinable with both hydrophilic and hydrophobic polymers in order to produce a compatible material with better properties than the individual components [7]. Despite all these benefits, zein like many other plant protein based biomaterials lacks sufficient mechanical strength and hydrolytic stability [5]. In order to improve their mechanical properties and water resistance, plant proteins can be blended with synthetic polymers or crosslinked [5,13].

In this study, neat zein and blends of zein-poly(glycerol sebacate) (PGS) were considered for developing fibers by electrospinning, following previous efforts by Dippold et al. [14]. In the study, the authors presented the first promising results for PGS prepolymer (PGS_P_)/zein blends with increasing zein content in the blends. PGS was chosen as a blend material due to its excellent mechanical properties, finely tunable degradation rate in biologically relevant media and biocompatibility [14,15,16]. However, owing to its low viscosity, electrospinning of neat PGS into fine fibers is difficult to achieve. Therefore, the blending of the natural polymer zein and the synthetic polymer PGS can be beneficial for both materials. In addition to the previous work [14], where 6:1, 5:1 and 4:1 ratios of zein-PGS_P_ were investigated, this study focuses on the fabrication and characterization of 6:1, 3:1 and 1:1 ratios of zeinPGS prepolymer as well as zein-mildly cross-linked PGS prepolymer (PGS_MXL_) blends. It was shown that a longer curing time increases the tangent modulus of PGS [17], which could be beneficial for improving the mechanical properties of the resulting blend fibers. Furthermore, the higher degree of cross-linking should lower the degradation rate of PGS in vitro [18]. However, as a result of the limited solubility of fully cross-linked PGS in organic solvents [19,20], partially cross-linked PGS was used for the fabrication of zein-PGS fiber mats in this study. All fiber mats were produced using less toxic solvents such as acetic acid (AcOH) or ethanol (EtOH) to enhance environmental friendliness during the fabrication step as well as the scaffolds’ biocompatibility [21]. In contrast to commonly used solvents for electrospinning, for example dichloromethane, chloroform, tetrahydrofuran, etc., AcOH and EtOH are classified as class 3 solvents according to the International Conference on Harmonisation of Technical Requirements for Registration of Pharmaceuticals For Human Use guidelines, which are considered to have a low toxic potential to man [22]. It was found that the electrospinning continuity of acetic acid is enhanced in contrast to ethanol, which might be attributed to a lower vapor pressure of AcOH and therefore, to a reduced amount of solvent, which evaporates at the needle tip [23]. Hence, AcOH was chosen for the fabrication of the blends due to its higher yield in comparison to aqueous EtOH. The fiber mats were investigated regarding their morphological, physiochemical and degradation properties. A preliminary in situ cross-linking study of neat zein with zero-length cross-linking agents *N*-(3-Dimethylaminopropyl)-*N*′-ethylcarbodiimide (EDC)/*N*-Hydroxysuccinimide (NHS) was performed and promising results were obtained.

## 2. Materials and Methods

### 2.1. Solution Preparation

The electrospinning solutions of neat zein (Sigma Aldrich^®^, Munich, Germany) in acetic acid (AcOH) (99–100% GPR RECTAPUR^®^, VWR, Darmstadt, Germany) and 70 wt % ethanol (EtOH) (Sigma Aldrich^®^, Munich, Germany) were prepared by dissolving 26 wt % and 30 wt % of zein, respectively. The solutions of zein in acetic acid or ethanol were stirred for 15 min at room temperature prior to electrospinning.

**Figure 1:**
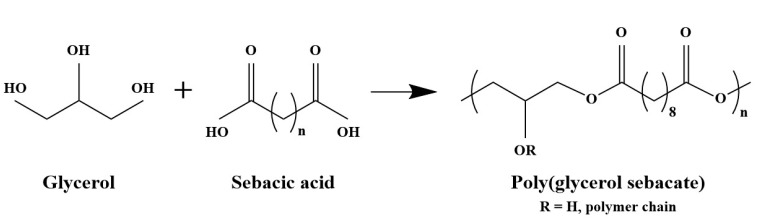
Poly(glycerol sebacate) obtained by polycondensation of glycerol and sebacic acid.

Zein-PGS_P_ and Zein-PGS_MXL_ fiber mats were prepared in 6:1, 3:1 and 1:1 ratios, each. For the fabrication of the blend solutions, PGS was conventionally synthesized according to the protocol of Wang et al. [15] (Figure 1). Briefly, an equimolar mixture of 0.1 M sebacic acid (99%, Sigma Aldrich^®^, Munich, Germany) and 0.1 M glycerol (BioXtra 99% (GC), Sigma Aldrich^®^, Munich, Germany) was heated at 120 °C under inert nitrogen atmosphere for 24 h to form the PGS prepolymer. Then, PGS_P_ was exposed to 120 °C under vacuum (1.3–2.5 × 10^−2^ mTorr) for a further 24 h and cured overnight to obtain mildly cross-linked PGS prepolymer. PGS_P_ and PGS_MXL_ were added to neat zein-acetic acid solution after 15 min stirring (total polymer concentration of 30 wt % for each composition). After 1.5 h of constant stirring the blend solutions were loaded into syringes for electrospinning.

For the first trial of *N*-(3-Dimethylaminopropyl)-*N*′-ethylcarbodiimide (EDC) (Sigma Aldrich, Munich, Germany) and *N*-Hydroxysuccinimide (NHS) (Sigma Aldrich, Munich, Germany) in situ cross-linked neat zein fibers, 26 wt % zein was dissolved in 90% aqueous EtOH. According to Kim et al. [24] 60 mg of the cross-linking reagents per gram of zein was added to the zein solution. The solution was stirred for 1.5 days at room temperature before electrospinning.

### 2.2. Electrospinning Process

A standard electrospinning setup (Starter Kit 40 KV Web, Linari srl, Valpiana (GR), Italy) was used for the fabrication of zein based fiber mats. The optimized electrospinning parameters were an applied voltage of 20 kV, a tip-target distance of 10 cm for neat zein and 15 cm for the blend, a flow rate of 0.4 mL/h and 0.7 mL/h for neat zein in AcOH or EtOH, respectively, and 0.3 mL/h for the blend. The environmental parameters, temperature and relative humidity (RH) were in a range of 23–26 °C and 36–64%, respectively. A summary of all electrospinning parameters can be found in Table 1. For comparison of different tip-to-target distances and RHs, a commercially available EC-CLI device (IME Technologies, Geldrop, The Netherlands) was used. The distance was varied between 10 cm and 15 cm and the relative humidity between 25% and 50%.

### 2.3. Characterization

*Scanning Electron Microscopy:* The morphology of the fiber mats was assessed by SEM analysis (FE-SEM, Auriga, Carl-Zeiss, Jena, Germany). The samples were investigated at magnifications ranging from 500 to 45000 X. Before SEM analysis, the samples were sputtered with gold using a Sputter Coater (Q150T, Quorum Technologies Ltd., Darmstadt, Germany). Average fiber diameters were calculated from measurements of 100 fibers from each sample using Fiji 1.51s analysis software (NIH, Bethesda, MD, USA) [25]. Minimal and maximum pore area of fiber mats were obtained using the mixed segmentation algorithm of DiameterJ [26] of Fiji 1.51s analysis software.

*Attenuated Total Reflectance Fourier Transform Infrared Spectroscopy:* FTIR analysis of all samples was carried out in attenuated total reflectance mode (ATR) using an FTIR spectrometer (Nicolet™ 6700, Thermo Scientific, Schwerte, Germany). In the measurement, 32 spectral scans were repeated over the wavenumber range of 4000 to 500 cm^−1^ with a resolution of 4 cm^−1^. The acquired data were visualized by Origin (OriginLab, Northampton, MA, USA).

*Mechanical Characterization*: The mechanical characterization of the fiber mats was performed by standard uniaxial tensile test (Z050, Zwick Roell AG, Ulm, Germany). The fibers were cut in strips with dimensions of 40 mm × 5 mm and arranged in paper frames to prevent unwanted stretching of the membrane before actual tensile testing. To record the stress-strain curves, samples were elongated at a speed of 10 mm/min with an initial length of 20 mm and 50 N cell load. The Young’s modulus (YM), ultimate tensile strength (UTS) as well as strain to failure (FS) was determined through the stress-strain curves, which were visualized by Origin. At least five specimens of each composition were tested to determine average values and standard deviations.

*Wettability*: In order to determine the wettability of the fiber mats, the static water contact angle of the samples was measured in air using the sessile drop method on a contact angle-measuring instrument DSA30 (Krüss GmbH, Hamburg, Germany). The fiber mats were fixed with microscope slides on both ends and droplets of 3 µL deionized water were placed onto the surface of the fiber mats at room temperature. At least five measurements at different positions on the same sample were carried out for each composition. The data was collected by the associated DSA software (DSA4 2.0, Krüss GmbH, Hamburg, Germany).

*In Vitro Degradation Study*: The in vitro degradation study of the electrospun fiber mats was carried out in phosphate buffered saline (PBS) (Sigma Aldrich^®^, Munich, Germany). The degradation behavior of the fiber mats was examined by three indicators, the morphology and chemical composition of the fiber mats after incubation and the pH variations of the degradation media. For each ratio the electrospun scaffolds were cut into squares with dimensions of 10 mm × 10 mm. The samples were incubated in 5 mL PBS at 37 °C for 1, 7 and 14 days with mild shaking (90 rpm) in a standard incubator (KS 4000 i Control, IKA, Staufen im Breisgau, Germany). Three specimens were tested for each time point. After the specific incubation period, the pH of the degradation medium was measured and the samples were removed from the solution. The samples were washed in deionized water and dried in a standard incubator for 24 h at 37 °C. Then, the morphologies and chemical composition were characterized by stereoscopic microscope, SEM and ATR-FTIR analyses, respectively. The pH change versus incubation time was recorded and plotted with Origin.

*Statistical Analysis*: All experimental data are presented as average values ± standard deviation. For structural analysis and mechanical testing, one-way analysis of variance (ANOVA, Origin) was used to analyze the differences between groups with a probability defined as (* *p* < 0.01).

## 3. Results and Discussion

Electrospinning of neat zein and zein-PGS solutions was successfully achieved except for Z1PGSp1 (50% RH), where only a film instead of fibers could be obtained. Observations of all distinct specimens on a macroscopic level revealed a homogeneous structure even if within all fiber mats splashes of solvent occurred. The removal of the fiber mats from the aluminum foil after electrospinning led to partial delamination of single layers within the fiber mats. This could affect the mechanical properties of the fiber mats as described later.

### 3.1. Fiber Morphology

It has been reported that the morphology and fiber diameter are strongly influenced by solvent and processing parameters, e.g., the polymer concentration, solvent factors, applied voltage, tip-target distance and flow rate, as well as ambient parameters [27]. All fiber mats were electrospun at 10 cm and 15 cm tip-to-target distance, but no significant change in the average fiber diameter and fiber morphology could be detected, which was also investigated and found by Miri et al. [28]. Therefore, neat zein fiber mats were electrospun at 10 cm distance, whereas the blends were electrospun at 15 cm distance, which was the working distance optimized by Dippold et al. [14]. When analyzing the influence of the electrospinning parameters on the fiber morphology, it was found that the relative humidity in the electrospinning chamber (25% and 50%) is the parameter, which affects not only the fiber morphology, but also the electrospinnability of the solutions and blends. 

**Figure 2:**
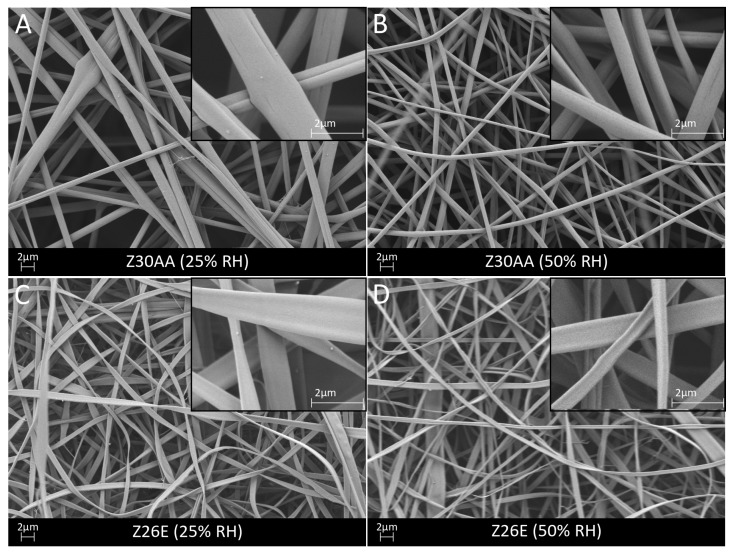
SEM micrographs of pure zein fibers using acetic acid (Z30AA) (**A**,**B**) and ethanol (Z26E) (**C**,**D**) as solvent at 25% and 50% relative humidity. The fibers are shown at 5000× magnification and at 20,000× magnification in the inserts.

Neat zein Z30AA demonstrated a defect- and bead-free smooth morphology of fibers in a fibrous and porous structure (Figure 2A,B) at 25% and 50% RH. Morphological assessment of the as-spun fibers revealed a circular cross section, unlike literature reports, where flat or ribbon-like morphologies were observed [14]. However, nearly every fiber was closely merged with another fiber causing almost doubling of the fiber diameter. The merging of fibers might appear if the fibers come in contact before all the solvent is evaporated. Altering of the surrounding environment, such as humidity, could influence the removal of solvent from the fiber surface and therefore affect the rate of solidification [29]. The average fiber diameter was determined as 1.1 ± 0.3 µm at 25% RH and 0.7 ± 0.2 µm at 50% RH whereby the diameter of merged fibers was considered as the fiber diameter of one fiber. Despite fusion of fibers, the average fiber diameter of zein fibers electrospun at 50% RH was slightly lower than reported in the literature for zein fibers [23,28]. The smaller fiber diameter could be explained by a much lower flow rate used in comparison to the literature [30], where a decrease in flow rate has been shown to lead to a decreased fiber diameter. In comparison to Z30AA, Z26E revealed a similar appearance on a macroscopic level but differed on a microscopic level. SEM micrographs showed that Z26E exhibited a ribbon-like morphology of the fibers (Figure 2C,D). The ribbon-like morphology was also observed by Selling et al. [23] when using aqueous ethanol as a solvent. The average fiber diameter of Z26E did not significantly differ from Z30AA being 0.8 ± 0.2 µm and 0.6 ± 0.3 µm at 25% RH and 50% RH, respectively. The results of the fiber morphology agree well with the results of Selling et al. [23] and Li et al. [27]. As also reported in the literature [23], using acetic acid as a solvent led to a higher yield in comparison to aqueous ethanol. Therefore, all blends of zein and PGS were fabricated using acetic acid.

**Figure 3:**
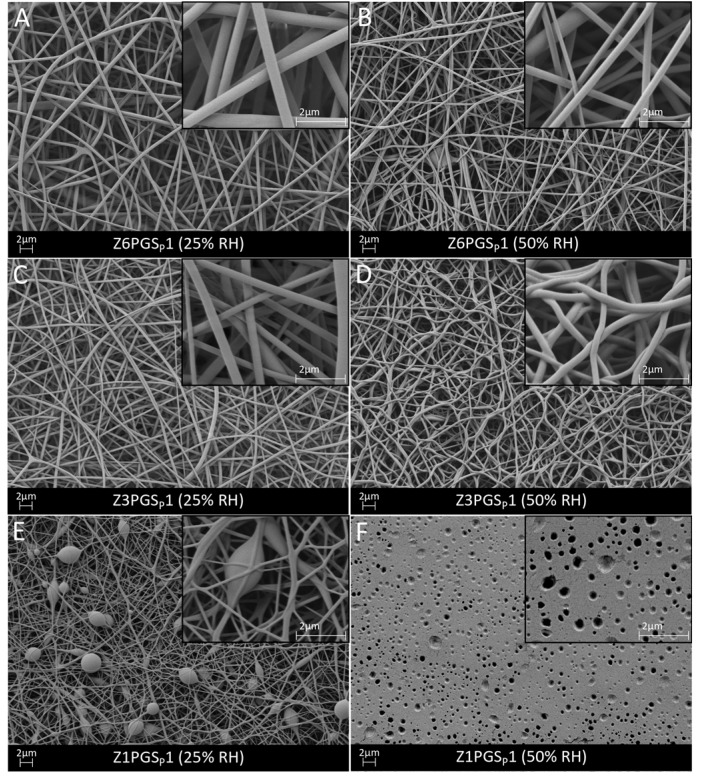
SEM micrographs of zein-PGS fibers: Z6PGS_P_1 (**A**,**B**), Z3PGS_P_1 (**C**,**D**) and Z1PGS_P_1 (**E**,**F**) at 25% and 50% relative humidity. The fibers are shown at 5000× magnification and at 20,000× magnification in the inserts.

For the blends Z6PGS_P_1 and Z3PGS_P_1, defect-free fibers were obtained at a low and high relative humidity, as shown in Figure 3A–D. Z6PGS_P_1 showed straight and non-agglomerated fibers, whereas with increasing PGS_P_ content more woven structures of fibers and agglomerated fibers were obtained at 50% RH in comparison to 25% RH. The agglomeration of fibers can occur due to incomplete evaporation of the solvent when the fibers reach the collector. This can be caused by the reduced viscosity according to the higher amount of PGS [19,30,31]. However, both blends showed smooth fibers and a homogenously distributed fiber diameter of 0.5 ± 0.1 µm (Z6PGS_P_1, 25% RH) and 0.3 ± 0.1 µm (Z3PGS_P_1, 25% RH; Z6PGS_P_1, 50% RH; Z3PGS_P_1, 50% RH). These values were in the range of values reported by Dippold et al. [14]. According to their results it was detected that with the increase of PGS content, the average fiber diameter decreased. Unlike the findings reported by Dippold et al. [14], where many beads were already observed for 4:1 ratio of zein-PGS, in this study bead-less fibers could also be obtained for the 3:1 ratio of zein-PGS. However, for Z1PGS_P_1, fibers with beads were obtained at 25% RH (0.2 ± 0.1 µm average fiber diameter) but no fibers at a higher relative humidity. Instead, a homogenous coating with closed nano-pores with an average diameter of 0.2 ± 0.1 µm was fabricated (Figure 3E,F). 

**Figure 4:**
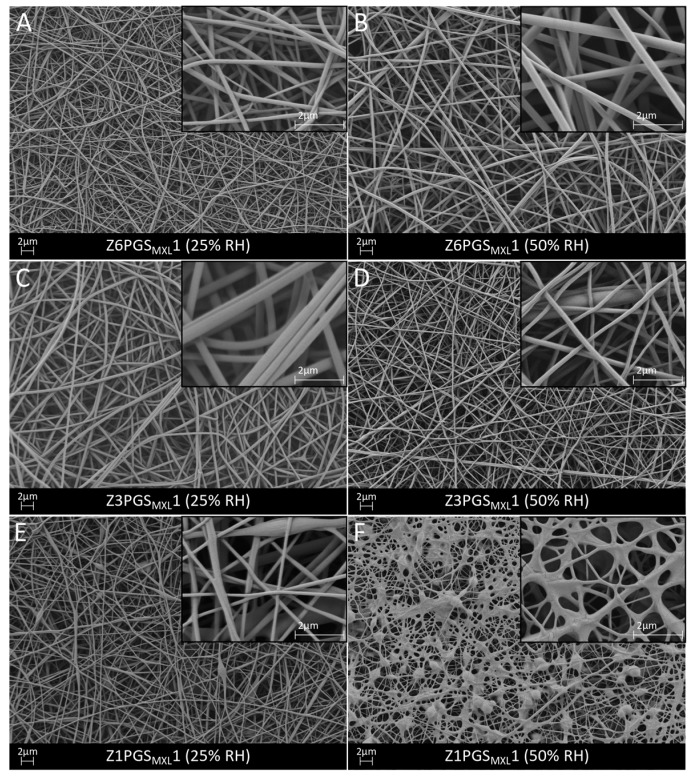
SEM micrographs of zein-PGS fiber mats: Z6PGS_MXL_1 (**A**,**B**), Z3PGS_MXL_1 (**C**,**D**) and Z1PGS_MXL_1 (**E**,**F**) at 25% and 50% relative humidity. The fibers are shown at 5000× magnification and at 20,000× magnification in the inserts.

As shown in Figure 4, fibrous and porous fiber mats were also observed for Z6PGS_MXL_1, Z3PGS_MXL_1 and Z1PGS_MXL_1. Blends of Z6PGS_MXL_1 and Z3PGS_MXL_1 showed round and smooth fibers but with increasing PGS_MXL_ content the fibers became more woven and some fibers merged. At a low relative humidity, the average fiber diameter was determined as 0.2 ± 0.1 µm (Z6PGS_MXL_1), 0.4 ± 0.1 µm (Z3PGS_MXL_1) and 0.2 ± 0.1 µm (Z1PGS_MXL_1). At 50% RH, it could be observed for PGS_MXL_ blended zein fibers that the fiber diameter decreased with increasing PGS_MXL_ content from 0.3 ± 0.1 µm (Z6PGS_MXL_1) to 0.2 ± 0.1 µm (Z3PGS_MXL_1), similar to PGS_P_ blended zein fibers. With even further increase of PGS_MXL_ content in Z1PGS_MXL_1, the average fiber diameter decreased to 0.09 ± 0.03 µm. However, at 50% RH many beads and junctions of fibers occurred in Z1PGS_MXL_1 blend, which could also be due to the reduced viscosity due to the higher amount of PGS, as already stated previously. In the following, the fiber mats electrospun at 50% RH were further evaluated due to the lower average fiber diameter in comparison to 25% RH.

### 3.2. Chemical Characterization

**Figure 5:**
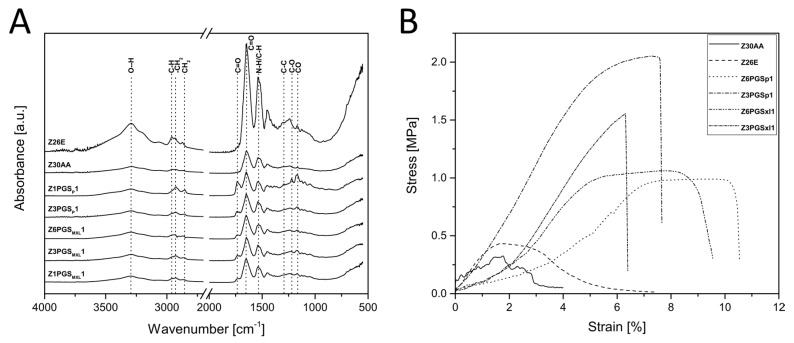
Physiochemical properties: (**A**) FTIR spectra of all produced fiber mats; (**B**) stress-strain curves of all fiber mats.

The obtained FTIR spectra of the distinct fiber mats are shown in Figure 5A. All characteristic bands of zein could be found in the spectra of neat zein as well as in the spectra of all blends. Peaks at ~1647 cm^−1^ and ~1539 cm^−1^ represent the characteristic vibrational bands of neat zein known as amide I and II vibration modes [32]. Amide I could be associated to C=O vibration stretching, whereas amide II is associated to both N–H bending and C–N stretching [32]. The broad band at ~3288 cm^−1^ was attributed to hydroxyl stretching vibrations [32]. Further characteristic bands of zein observed at ~2960 cm^−1^ and ~2928 cm^−1^ were considered to be C–H stretching from CH_3_ and CH_2_ functional groups derived from free fatty acids present in zein [32]. In the spectra of Z6PGS_P_1 and Z6PGS_MXL_1 typical peaks of PGS could already be observed. In the spectra, which were normalized between 0 and 1, it was found that the intensity of bands attributable to PGS was enhanced with increasing amounts of PGS in the blend. The broad peak centered around ~3400 cm^−1^ could be related to hydroxyl bond stretch vibration (O–H) of PGS and is overlapping with the peak of zein at ~3288 cm^−1^ [33,34]. With an increasing amount of PGS, this peak became broader. The bands at ~2926 cm^−1^ and ~2851 cm^−1^ were assigned to stretch vibrations of the alkyl group (–CH_2_) of PGS [15]. The signals at ~1732 cm^−1^ and ~1173 cm^−1^ exhibited the presence of ester (C–O) and carbonyl groups (C=O) of PGS [34]. In the spectra of Z1PGS_MXL_1, a further peak at ~1293 cm^−1^ arose, which can be attributed to backbone C–C and C–O stretching modes in PGS [33,35]. Furthermore, it could be detected that with an increasing amount of PGS, the peak at ~1242 cm^−1^ in Z30AA was shifted to lower wavenumbers at ~1221 cm^−1^ for Z1PGS_MXL_1, being a characteristic peak for C–O stretch vibrations of PGS [20].

### 3.3. Mechanical Characterization

The mechanical properties of the fiber mats were investigated by tensile strength tests. Studies have shown that scaffolds developed from proteins (e.g., zein) have poor mechanical properties, especially in the wet state [5,13]. Tensile testing of neat zein (Z30AA) revealed a Young’s Modulus (YM) of 22 ± 7 MPa, an ultimate tensile strength (UTS) of 0.3 ± 0.1 MPa and a failure strain (FS) of 2.7 ± 0.9%. Similar values for the YM and UTS were obtained by Yao et al. [36], whereas they reported a much lower value for the FS. These poor mechanical properties could mainly be attributed to the weak bonding forces between different layers, as expressed by the delamination of layers during the tensile test. The addition of PGS_P_ and PGS_MXL_ to zein led to an increase in the UTS and FS, whereas the YM did not significantly change. Detailed values can be found in Table 2. The high standard deviation could be caused by the delamination process during tensile strength test. Testing freshly electrospun fiber mats, it was found that all zein containing blends were elongated to a certain amount until a smooth delamination of different layers appeared on the specimens. Otherwise, if the fiber mats had been stored for one month at room temperature, no delamination process within the specimens occurred, moreover, the fiber mats broke with a detectable noise. The fiber mats became thus more brittle and much lower mechanical properties were achieved indicating that the storage time might alter the zein fiber composition. This could explain the lower failure strain of Z30AA, Z3PGS_P_1 and Z3PGS_MXL_1 in comparison to values reported in literature. In contrast to the results of this study, Dippold et al. [14] reported a decrease of mechanical properties with a decreasing amount of zein to final values of YM of 14 ± 3 MPa, UTS of 13 ± 0.1 MPa and FS of 5 ± 3% for a 6:1 ratio of zein to PGS_P_. All stress-strain graphs can be found in Figure 5B. 

### 3.4. Wettability

The hydrophilic or hydrophobic behavior of the material affects the protein adsorption as well as cell adhesion on the material. The wettability of the as-spun fiber mats was evaluated by contact angle measurements. Being an amphiphilic protein, zein possesses hydrophobic as well hydrophilic properties [7]. However, neat zein and zein containing fiber mats were completely wetted immediately after the water drop was placed onto the surface. This phenomenon could probably be explained by the poor morphological stability of zein if fabricated into fibers as well as the presence of PGS in the blends. A similar behavior was also observed by Dippold et al. [14] and Ali et al. [37]. Other studies have shown that zein containing fiber mats shrink in contact with aqueous media and lose their fibrous structure becoming a film [37,38]. It could be assumed that the highly hydrophilic behavior is favorable for cell attachment and proliferation [39] but the shrinking behavior and morphological instability of zein and its blends limit their specific application in the human body.

### 3.5. Degradation Behavior

Zein, consisting of polypeptides, is biodegradable. Wang et al. [40] have shown that porous zein scaffolds completely degrade within eight months in vivo, where they undergo mostly enzymatic degradation. On the other hand, PGS degrades relatively fast via hydrolysis of its ester linkages depending on its cross-linking level [41]. It was found that cross-linked PGS is resorbed within 60 days in vivo [42], where it undergoes surface degradation mainly caused by cleavage of ester linkages [41]. 

**Figure 6:**
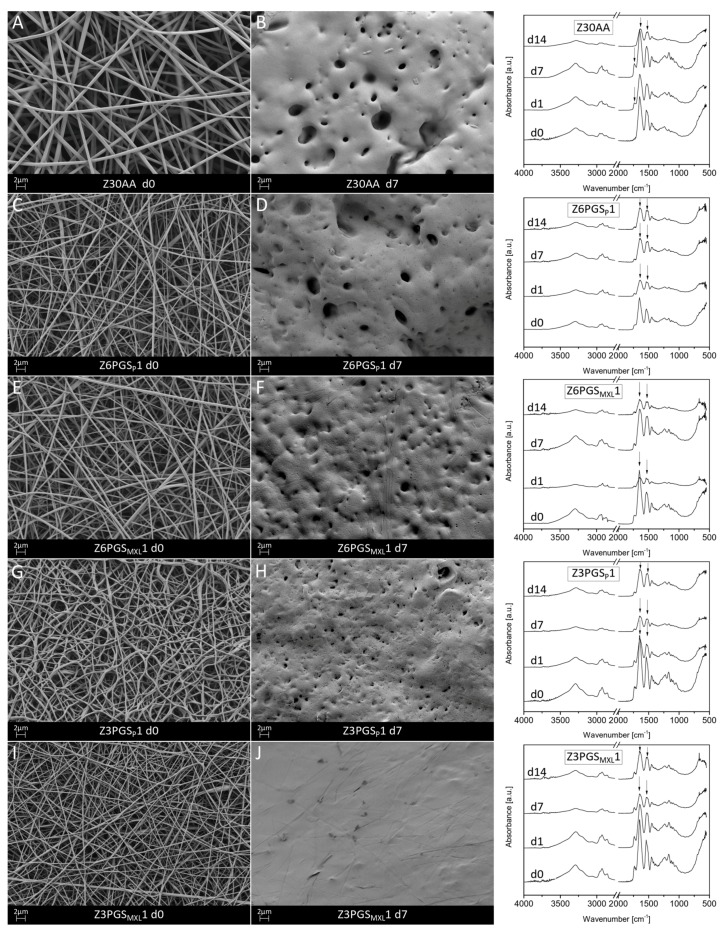
SEM micrographs of Z30AA (**A**,**B**), Z6PGS_P_1 (**C**,**D**), Z6PGS_MXL_1 (**E**,**F**), Z3PGS_P_1 (**G**,**H**) and Z3PGS_MXL_1 (**I**,**J**) before and after 7 days of degradation at 5000× magnification and respective FTIR spectra (the peaks in the FTIR spectra are discussed in the text).

The incubated samples were investigated regarding their morphological and chemical structure after incubation times of 1, 7 and 14 days in PBS. To assess the chemical nature of the degradation products, the pH of the incubation medium was also studied. Figure 6 shows the fiber mats before and after 7 days of immersion as well as their FTIR spectra at specific time points. In previous experiments, neat zein (Z30AA) as well as all blends of zein and PGS lost their morphological stability and showed a significant decrease in surface area after 1 day in PBS. On a microscopic level, it was found that, already starting at day 1, the fibrillar structure of the fiber mat was almost lost. At day 14 many areas of the fiber mat lost their fibrous structure and became film-like. Xu et al. [43] suggested that this film-forming process happens due to a higher mobility of polymer chains in water since water is a plasticizer of zein. This allows electrospun zein fiber mats to reduce their surface energy by forming a film and explains the shrinkage of zein in aqueous solutions. For all blends it could be detected that the fibrillar structure was already lost after 1 day of immersion. For high PGS content and mildly cross-linked PGS it was found that the contour of some original fibers was still detectable up to 14 days. However, in cross sections, fiber mats made by the blend revealed the loss of the fibrillar structure, forming a fully densified film.

FTIR analysis of neat zein revealed a new peak at around 1739 cm^−1^ after 1 and 7 days, which could be attributed to C=O stretch vibrations due to the presence of ester groups in methylated zein [44]. This peak completely disappeared after 14 days of immersion. Furthermore, after 14 days the spectra of neat zein showed a decrease of the peak around 3288 cm^−1^, which was attributed to hydroxyl stretch vibrations [32], leading to the assumption that slight hydrolysis of zein has occurred. In all blends of zein-PGS it could be detected that the sharp peaks attributable to amide I (1650 cm^−1^) and amide II (1539 cm^−1^) vibration modes became broader. However, no significant changes concerning the characteristic peaks of PGS were detected. In particular, since the broad absorption band centered at around 3400 cm^−1^ corresponding to PGS overlaps with the broad band at 3288 cm^−1^ assigned to hydroxyl stretching of zein, it could not be stated which component was degraded.

**Figure 7:**
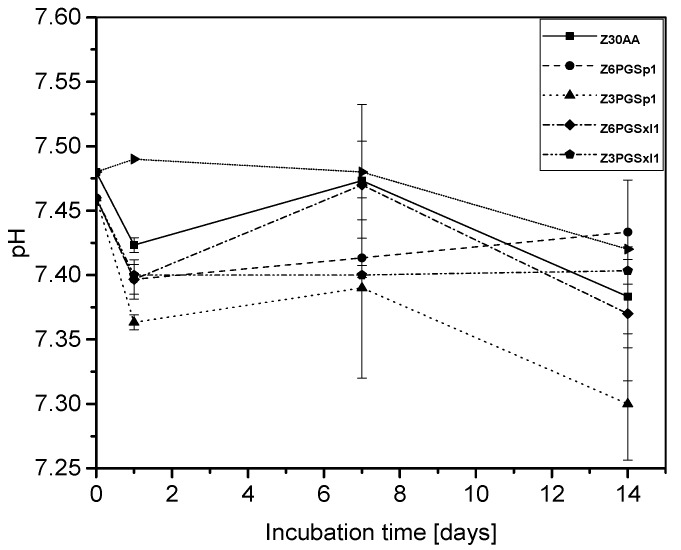
pH change of the different degradation media of neat zein and zein-PGS fiber mats at specific time points.

Generally, the pH of neat zein lies in the alkaline range but varies depending on its amino acid composition [45], whereas PGS, when degrading, provides an acidic character to the degradation medium due to release of carboxylic groups caused by ester cleavage and due to the presence of unreacted carboxylic acid groups on the PGS backbone [41]. As presented in Figure 7, after a first drop of pH at day 1, pH measurements showed an increase until day 7, which could be attributable to changes of zein dominating the degradation behavior of the blend. After 14 days of immersion, it was found that the ratio with the highest amount of non-cross-linked PGS, namely Z3PGS_P_1, exhibited the largest drop of pH to around 7.30 ± 0.04. In comparison to this, all the other ratios ended in a similar range of pH after 14 days. Additionally, it was detected that the ratio with the lowest amount of mildly cross-linked PGS (Z6PGS_MXL_1) followed the pH change of neat zein most closely.

As also shown in the literature [13,37,38], the morphological instability of zein fiber mats in aqueous solutions was proven by the collapse into films, whereby the surface area of fiber mats, as well as the number of interconnected pores were considerably decreased. It has been found that this behavior is also linked to a decrease in tensile strength [5], which could be limiting for application in the body. Whereas it is expected that zein undergoes enzymatic degradation in vivo [40] and is not affected in the absence of enzymes [46], the morphological changes, changes in the IR spectra as well as changes in the pH of the degradation medium, indicates that neat zein underwent slight hydrolytic degradation in PBS. Dippold et al. [14] concluded that zein likely reacts via hydrolysis with PBS solution as it was dissolved in acetic acid and blended with PGS. Furthermore, they suggested that the zein component of the blend fiber mat was released from the fibers during immersion in the degradation medium. Results showed that mildly cross-linked PGS was more stable against hydrolysis than uncross-linked PGS. Overall, it can be summarized that initially PGS was degraded, and the fibrillary structure collapsed into a film-like structure due to the zein component in the blend. Then, zein outweighed the degradation rate of PGS. Finally, between 7 and 14 days of immersion the degradation rates of both components seemed to be equal.

### 3.6. In Situ Cross-Linking of Fibers

**Figure 8:**
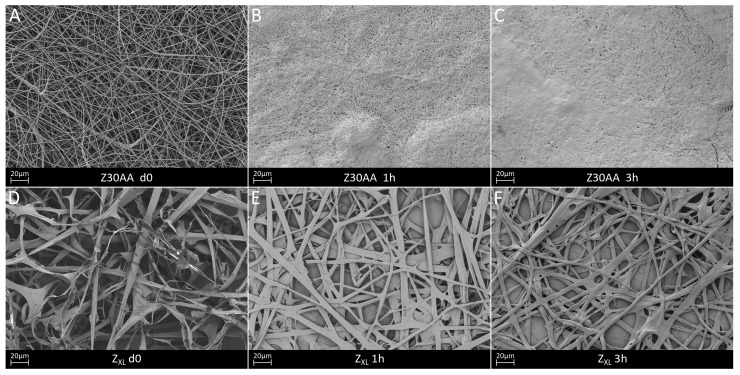
SEM micrographs of uncross-linked Z26E fiber mats (**A**–**C**) and EDC/NHS cross-linked Z_XL_ fiber mats (**D**–**F**) before and after 1 and 3 h of PBS incubation.

To overcome the limited stability of zein fibers in aqueous media, neat zein was cross-linked with EDC/NHS for 1.5 days. Kim et al. [24] have shown that the mild cross-linking reagents EDC and NHS are able to form intermolecular amide bonds in zein solutions. They reported that EDC connects carboxyl groups in one protein molecule to amine groups in the other protein molecule. Simplified, by the reaction of the carboxylic groups with EDC, a highly activated ester intermediary form (*O*-acylurea) is created. This ester derivative reacts with the primary nitrogen nucleophiles of the amino compound and leads to the formation of the amino linkage with the release of the soluble substituted urea. NHS stabilizes the activated ester and thereby enhances the coupling efficiencies of carbodiimides for conjugating carboxylated compounds with primary amines [24,47]. The cross-linked fibers revealed a more ribbon-like, flat structure and the fiber thickness increased significantly to 4 ± 2 µm in comparison to Z26E (Figure 8A,D). After immersion of the uncross-linked and cross-linked zein fiber mats for 1 h and 3 h in PBS, the EDC/NHS cross-linked zein fibers maintained their fibrous structure, whereas the uncross-linked Z26E fibers started to swell and fuse together. In comparison, after 3 h in PBS, Z26E was a densified, low porous structure and in some areas the fibrous structure was already completely lost. Even if the initial fiber diameter was significantly higher than the fiber diameter of Z26E, the cross-linked fibers did not swell. Their fiber diameter remained constant after 3 h (4 ± 1 µm).

**Figure 9:**
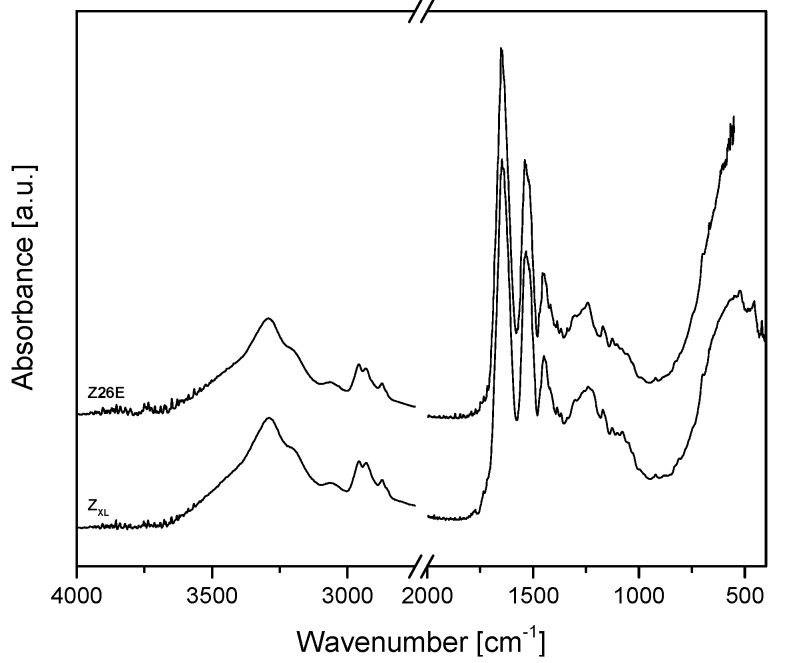
FTIR spectra of Z26E and cross-linked zein.

The FTIR spectra of uncross-linked and cross-linked zein are shown in Figure 9. No differences in the FTIR spectra of cross-linked in comparison to uncross-linked zein was found. As stated previously, during cross-linking amide bonds are formed, which are overlapping with existing amide bonds and therefore they are not detectable in the FTIR spectra.

## 4. Conclusions

In this study, fiber mats from neat zein and zein-PGS blends in 6:1, 3:1 and 1:1 ratios were obtained by electrospinning. Except for the 1:1 ratio of zein to PGS prepolymer, all compositions were fabricated successfully and SEM observations revealed homogeneous fiber mats. It was found that the relative humidity influenced the electrospinnability of the blends. At lower relative humidity (25% RH), all blends were electrospinnable, however, the average fiber diameter was slightly higher than at 50% RH. The fiber diameter of neat zein in acetic acid or ethanol was determined to be 0.7 ± 0.2 µm and 0.6 ± 0.3 µm, respectively. The addition of PGS to zein and its increase in percentage decreased the fiber diameter from around 0.3 µm to 90 nm. Bead-less fibers could be successfully fabricated also for blends with a high percentage of PGS (3:1 zein-PGS). Regarding a 1:1 ratio of zein-PGS, mildly cross-linked PGS prepolymer showed better electrospinnability in comparison to PGS prepolymer. ATR-FTIR analysis of the different fiber mats revealed the characteristic absorption bands of each polymer in the fibers and a successful mildly cross-linking of PGS. Tensile strength tests showed that the addition of PGS to zein did not significantly affect the Young’s modulus, but the ultimate tensile stress and failure strain were increased in comparison to the neat zein fiber mats. Due to the rapid immersion of the water drop into the fiber mats of all blend ratios, the wettability of the fiber mats could not be measured. Degradation tests of the different uncross-linked fiber mats indicated that the fibrous structure was already lost after 1 day of immersion, leading to a film-like structure mainly due to the morphological instability of zein in contact with aqueous solutions. To overcome this limitation, zein was cross-linked using the zero-length cross-linking reagents EDC and NHS for 1.5 days prior to electrospinning. Preliminary results showed a better aqueous stability than uncross-linked zein fibers. The cross-linking of zein and zein-PGS fibers with these mild cross-linking agents should be optimized and closely investigated in future with regard to mechanical properties, degradation rate and cytocompatibility. However, the novel type of electrospun nano-fibrous zein patches incorporating the synthetic biopolymer PGS show suitable properties for application in soft tissue engineering. Although they need further optimization, the morphological properties of the fiber mats are appropriate for several soft tissue engineering applications and the mechanical properties are even higher than properties needed for cardiac tissue engineering, for example. Hence, the blending of zein with PGS is promising, especially with mildly cross-linked PGS prepolymer, which has not been investigated before.

## Figures and Tables

**Table 1:** Compositions of polymer solutions and electrospinning parameters of all fiber mats fabricated in this study.

Sample	Polymers/Ratio	Solution Concentration/Solvent System	Voltage (kV)	Tip-Target Distance (cm)	Flow Rate (mL/h)
Z30AA	Zein	30 wt % in AcOH	20	10	0.4
Z26E	Zein	26 wt % in EtOH	20	10	0.7
Z6PGS_P_1	Zein:PGS_P_ 6:1	30 wt % in AcOH	20	15	0.3
Z3PGS_P_1	Zein:PGS_P_ 3:1	30 wt % in AcOH	20	15	0.3
Z1PGS_P_1	Zein:PGS_P_ 1:1	30 wt % in AcOH	20	15	0.2–0.3
Z6PGS_MXL_1	Zein:PGS_MXL_ 6:1	30 wt % in AcOH	20	15	0.3
Z3PGS_MXL_1	Zein:PGS_MXL_ 3:1	30 wt % in AcOH	20	15	0.3
Z1PGS_MXL_1	Zein:PGS_MXL_ 1:1	30 wt % in AcOH	20	15	0.3
Z_XL_	Zein	26 wt % in EtOH	20	10	0.3

**Table 2:** Average fiber diameter, pore area and mechanical properties of the distinct fiber mats.

Sample	Average Fiber Diameter (µm)	Pore Area, Min–Max (µm^2^)	Young’s Modulus (MPa)	Ultimate Tensile Strength (MPa)	Failure Strain (%)
Z30AA	0.7 ± 0.2	0.02–18.3	22 ± 7	0.3 ± 0.1	2.7 ± 0.9
Z26E	0.6 ± 0.3	0.02–15.6	19 ± 9	0.4 ± 0.03	5 ± 1
Z6PGS_P_1	0.3 ± 0.1	0.01–10.6	7 ± 2	1.0 ± 0.1	10 ± 1
Z3PGS_P_1	0.3 ± 0.1	0.01–8.7	32 ± 10	2.2 ± 0.8	6 ± 2
Z1PGS_P_1	-	-	-	-	-
Z6PGS_MXL_1	0.3 ± 0.1	0.01–10.3	24 ± 17	1.2 ± 0.2	11 ± 4
Z3PGS_MXL_1	0.2 ± 0.1	0.01–6.2	25 ± 16	1.4 ± 0.3	5 ± 1
Z1PGS_MXL_1	0.09 ± 0.03	0.01–5.0	-	-	-
